# Emergency Surgery for Adnexal Torsion in Late Preterm Pregnancy Causing Term Vaginal Delivery: A Case Report and Literature Review

**DOI:** 10.7759/cureus.64268

**Published:** 2024-07-10

**Authors:** Kentaro Taniguchi, Yuji Tanaka, Tsukuru Amano, Shunichiro Tsuji, Takashi Murakami

**Affiliations:** 1 Department of Obstetrics and Gynecology, Shiga University of Medical Science, Otsu, JPN

**Keywords:** laparotomy, emergency surgery, literature review, case report, paratubal cyst, fallopian tube torsion, late-preterm, vaginal delivery, pregnancy, adnexal torsion

## Abstract

Adnexal cyst torsion in late preterm pregnancies is rare, but it frequently causes secondary uterine contractions. Thus, deciding on performing a simultaneous cesarean section due to the potential for early postoperative labor onset is crucial despite no obstetric indications. Here, we report a case of adnexal torsion at 34 weeks of gestation treated with emergency surgery, followed by a full-term vaginal delivery, along with a literature review. A 31-year-old primigravida at 34 weeks and four days of gestation presented to the emergency department with right lower abdominal pain. An emergency laparotomy was performed to achieve term delivery, suspecting right ovarian cyst torsion without signs of fetal distress. General anesthesia with sevoflurane was selected over spinal anesthesia, considering the incision height. The patient was placed in the left lateral decubitus position on the operating table to ensure proper visualization and maintain uterine circulation. A 4-cm transverse skin incision was made under ultrasound guidance, revealing the twisted right paratubal cyst immediately beneath. The cyst was excised, and the torsion was relieved. The postoperative course was uneventful, and spontaneous labor occurred at 39 weeks and six days of gestation, resulting in a vaginal delivery at 40 weeks. This case demonstrates that even late preterm adnexal torsion can be managed safely with appropriate surgical techniques, allowing for a subsequent term vaginal delivery.

## Introduction

Adnexal cysts during pregnancy undergo torsion, with reports indicating a fivefold increase in the risk during pregnancy [1]. A study of 174 patients with known adnexal tumors revealed 24 (13.8%) tumor torsions [2]. Torsion is more frequent in the first and second trimesters, whereas it is relatively rare in the third trimester, occurring in 5-10% of cases [2,3]. The frequency is even lower when focusing on late preterm. Secondary uterine contractions may occur when adnexal torsion occurs in late preterm pregnancy, causing the potential onset of spontaneous labor immediately postoperatively. Therefore, performing a cesarean section alongside the surgery for torsion is a treatment option even in the absence of obstetric indications such as fetal distress. However, the goal should be to achieve term delivery whenever possible [4]. Several challenges must be addressed, including the difficulty of obtaining a clear view due to the enlarged pregnant uterus and the issue of suppressing secondary uterine contractions, when performing surgery solely for adnexal torsion in late preterm pregnancy.

Here, we report a patient with a pregnancy complicated by fetal heart anomaly who experienced adnexal torsion at 34 weeks of gestation. An emergency laparotomy was performed to achieve term vaginal delivery, and the patient subsequently delivered vaginally at 39 weeks. Additionally, we conducted a literature review of term or late-preterm adnexal torsion cases by searching PubMed for articles published up to June 20, 2024.

## Case presentation

A 31-year-old primigravida with no significant medical or family history was undergoing prenatal care at our hospital due to a fetal congenital heart anomaly. The patient was under the care of another hospital during the first trimester, where the presence or absence of adnexal cysts was not documented. The patient presented to the emergency department with right lower abdominal pain at 34 weeks and four days of gestation. Her vital signs were stable. A vaginal examination revealed a 1.5-cm cervical dilation. Cervical length was 28 mm. Blood test results indicated no increase in inflammation (Table 1).

**Table 1:** Blood sampling data at presentation with abdominal pain

		Result	Reference Range
Hemoglobin	(g/dL)	12.2	11.6–14.8
White cell count	(x 10 ^9^ /L)	11.0	3.3–8.6
Platelets	(x 10 ^9^ /L)	33.9	31.7–35.3
C-reactive protein	(mg/L)	1.01	0.00–0.14
Urea	(mmol/L)	4.1	8.0–20.0
Creatinine	(mg/dL)	0.42	0.46–0.79
Sodium	(mmol/L)	136	138–145
Potassium	(mmol/L)	3.8	3.6–4.8

**Figure 1:**
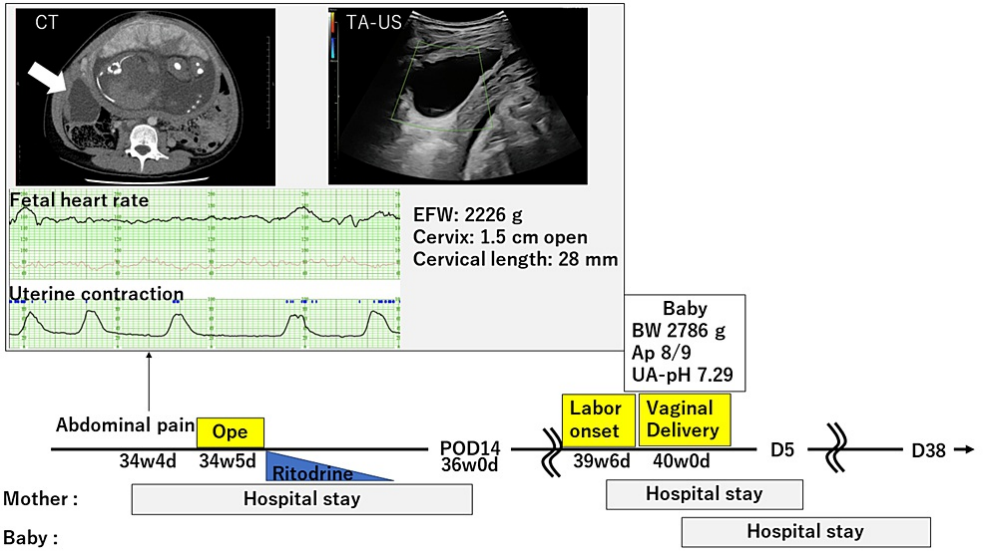
Schemas of the course The white arrow indicates the torsion right adnexa. EFW: estimated fetal weight; CT: computed tomography; TA-US: transabdominal ultrasound; POD: postoperative day; D: post-admission day; BW: body weight; Ap: Apgar score; UA-pH: umbilical cord blood artery pH

The non-stress test revealed a reassuring fetal heart rate pattern, with irregular uterine contractions occurring every two to six minutes. The estimated fetal weight was 2226 g. Transabdominal ultrasound revealed no oligohydramnios, placental thickening, nor placental hematoma but determined a cystic mass of >5 cm in diameter on the right posterior side of the uterus, which was tender upon palpation. Contrast-enhanced computed tomography (CT) revealed a cystic tumor on the right side of the uterus measuring 93 mm × 60 mm × 73 mm and poor contrast enhancement of the right ovarian vein, indicating right ovarian cyst torsion. We decided to perform only a tumor resection via laparotomy to achieve term delivery (Figure 1).

**Figure 2:**
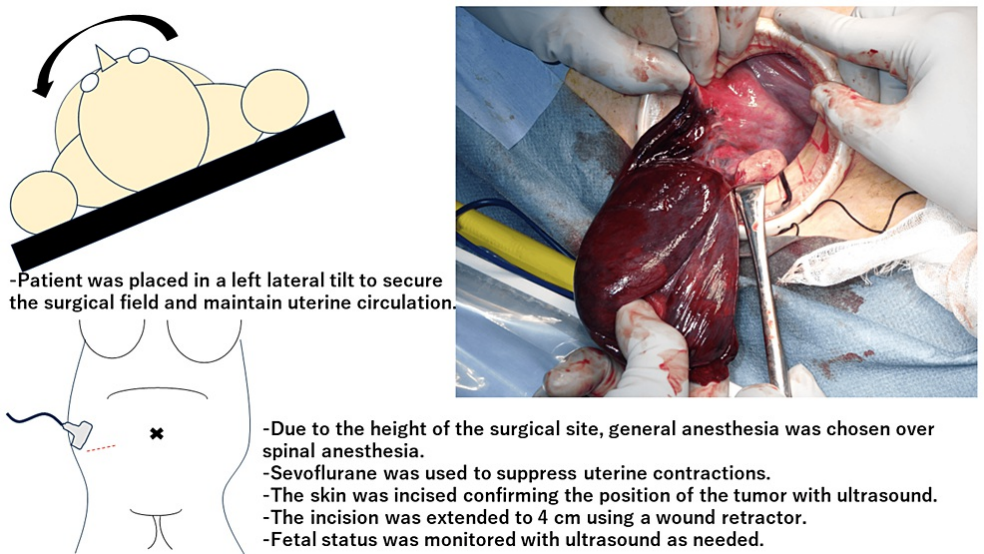
Surgery and perioperative management

The surgery lasted 52 minutes with minimal blood loss. Fetal heartbeat was confirmed before and after the surgery, and no cervical shortening was observed. A transabdominal ultrasound confirmed the position of the tumor, marking the skin directly above. General anesthesia with sevoflurane was selected over spinal anesthesia, considering the height of the surgical site. The patient was placed in a left lateral tilt position to secure the surgical field and maintain uterine circulation. A 4-cm transverse skin incision was made under ultrasound guidance, and a plastic wound retractor was used to access the abdominal cavity, where a paratubal cyst on the right side was determined. The cyst was severely twisted and was excised. Bipolar coagulation was used to achieve hemostasis. An anti-adhesion barrier was applied to the right adnexa, confirming the fallopian tube preservation and the absence of other abnormalities, and the incision was closed (Figure 2).

Ritodrine hydrochloride was gradually reduced from 100 μg/minute and discontinued 10 days postoperatively. The patient was discharged home in good condition 14 days postoperatively. The remainder of the pregnancy was uneventful, and labor began spontaneously at 39 weeks and six days, resulting in a vaginal delivery at 40 weeks. The neonate weighed 2786 g, with Apgar scores of 8 and 9 at one and five minutes, respectively, umbilical artery pH of 7.288, and BE of -7. Postnatally, the neonate was admitted to the neonatal intensive care unit and diagnosed with the same complex congenital heart defects (double outlet right ventricle, major aortopulmonary collateral artery, and left heart hypoplasia) identified prenatally. The infant was discharged on day 38 and later diagnosed with 22q11.2 deletion syndrome via fluorescence in situ hybridization (FISH).

## Discussion

A literature review identified 12 cases of adnexal torsion from late preterm to term [5-15], including our case. Of these, nine (75%) cases involved the right side, whereas nine (75%) cases were paratubal cysts. Indications for cesarean section were unclear in many cases, but only three (25%) cases resulted in vaginal delivery (Table 2).

**Table 2:** A review of cases requiring emergency surgery for late preterm and term torsion of adnexal cysts R: right; L: left; FT: fallopian tube; OV: ovary; CS: cesarian section; VD: vaginal delivery; N/A: not applicable

Author	Age	History of CS	Gestational weeks at adnexal torsion	Side	Site of torsion	Size of adnexa	Histopathology	Treatment	Delivery
Current case	31	0	34 + 4	R	FT	9 cm × 6 cm × 7 cm	Paratubal cyst	Cystectomy	39 + 6 weeks VD
Koumoutsea et al. [5]	33	2	35 + 2	R	Ov	6 cm × 3 cm	Dermoid cyst	Oophorocystectomy and CS	35 + 2 weeks CS
ten Cate et al. [6]	33	N/A	35 + 4	L	FT	4.4 cm	N/A	Detorsion and CS	35 + 4 weeks CS
Chohan et al. [7]	33	0	35 + 4	R	FT	9 cm × 6cm × 4 cm	N/A	Laparoscopic drainage of the paratubal cyst	38 weeks VD
Park [8]	36	1	35 + 6	R	FT	4 cm × 3 cm	Hydrosalpinx	Salpingectomy and CS	36 weeks CS
El-agwany [9]	26	2	35	R	Ov	15 cm × 10 cm	Paraovarian cyst	Oophorocystectomy and CS	35weeks CS
Zhan et al. [10]	28	0	35	R	Ov	10 cm × 9 cm × 6 cm	Mucinous cystadenoma	Laparoscopic oophorocystectomy	40 + 2 weeks VD
Sun et al. [11]	32	0	36 + 4	R	FT	7 cm × 5 cm	Necrosis of the FT	Partial salpingectomy and CS	37 + 1 weeks CS
Simsek et al. [12]	27	0	36	R	FT	3 cm × 3 cm	Necrosis of the FT	Salpingectomy and CS	37 weeks CS
Bakacak et al. [13]	18	0	37	R	FT	5 cm × 2 cm × 3 cm	Necrosis of the FT	Salpingectomy and CS	37 weeks CS
Thiyagalingam et al. [14]	41	1	37	L	Ov	10 cm × 10 cm	Necrosis of the adnexa	Salpingo-oopherectomy and CS	37 weeks CS
Gulino et al. [15].	23	0	38+5	L	FT	6.5 cm × 5 cm	Necrosis of the FT	Salpingectomy and CS	38 + 5 weeks CS

Managing adnexal torsion in late preterm is difficult due to secondary uterine contractions. If only adnexal surgery is performed, the risk of spontaneous labor in the immediate postoperative period means that a concurrent caesarean section may be considered even in cases where there are no obstetric indications. Nevertheless, instead of opting for cesarean delivery in preterm births, it is preferable to aim for vaginal delivery at full term whenever possible [4]. In this case, we aimed for term vaginal delivery and planned only adnexal surgery. The presence of fetal heart anomalies further supported this decision, but term delivery should be pursued even without such anomalies. The optimal interval between laparotomy during pregnancy and subsequent vaginal delivery is unclear, but studies reported vaginal delivery four weeks post laparotomy [16] and 18 days post laparoscopy [7]. Vaginal delivery was safely achieved five weeks postoperatively in the current case.

Technical difficulties in performing surgery for ovarian cysts in late preterm pregnancy include visualizing the surgical field due to the enlarged uterus and managing secondary uterine contractions. We rotated the patient to the left to secure the surgical field for a right ovarian cyst torsion. This technique protects uterine blood flow, as previously reported [17,18]. This technique may be beneficial for visualization, considering the higher incidence of right-sided torsion in our review. Preoperative ultrasound marking of the tumor location enabled direct, small incision access. High skin incision levels required general anesthesia, and sevoflurane was selected for its superior uterine contraction inhibition. We used a plastic wound retractor to maintain a clear view through the small incision and administered ritodrine pre- and postoperatively. The most prevalent types of adnexal masses in pregnancy that require surgical management are dermoid cysts (32%), endometriomas (15%), functional cysts (12%), serous cystadenomas (11%), and mucinous cystadenomas (8%) [19]. However, our literature review revealed that dermoid cysts, endometriomas, and functional cysts were not predominant in late pregnancy. Our literature review indicated a higher occurrence of paratubal cyst torsion, which typically involves less bleeding and simpler surgery than ovarian cysts, thereby potentially supporting the avoidance of cesarean sections without obstetric indications.

Regarding the size of adnexal tumors, those with a diameter of 6-8 cm demonstrate a higher rate of torsion compared to other sizes (22% vs. 9%, odds ratio: 2.8 (95% confidence interval: 1.1-6.6)) [2]. Furthermore, our review shows that adnexal tumors of this size are more likely to undergo torsion even in late preterm or term pregnancy. Elective laparoscopic surgery in the early stages of pregnancy may help to prevent torsion if an adnexal cyst of this size is detected early.

A limitation of this study is the open surgery approach. Laparoscopic surgery in late pregnancy remains debated, but almost all late preterm torsion cases in the literature involve laparotomy. However, Cohen et al. revealed successful laparoscopic surgery in 27-38 weeks of gestation, including two late preterm cases, with one subsequent vaginal delivery [17]. They emphasized techniques such as port placement adjustment, patient rotation, and intraoperative CO2 monitoring to maintain low intra-abdominal pressure and physiological alignment. We opted for open surgery while an initial laparoscopic evaluation followed by laparotomy if needed could have been considered.

## Conclusions

The potential development of this study depends on its application. Surgery might be performed for adnexal torsion even in early-term pregnancy using the techniques described here, which might lead to full-term vaginal delivery. Additionally, a staged approach enables cesarean delivery at term in cases of late preterm with a history of cesarean section, if the patient accepts the presence of two surgical scars. Therefore, adnexal torsion tumors in the late stages of pregnancy are safely managed with careful surgical techniques, allowing for subsequent full-term vaginal delivery.
